# Empagliflozin, calcium, and SGLT1/2 receptor affinity: another piece of the puzzle

**DOI:** 10.1002/ehf2.12345

**Published:** 2018-07-19

**Authors:** Stefan D. Anker, Javed Butler

**Affiliations:** ^1^ Division of Cardiology and Metabolism, Department of Cardiology (CVK), Berlin‐Brandenburg Center for Regenerative Therapies (BCRT), German Centre for Cardiovascular Research (DZHK) partner site Berlin Charité Universitätsmedizin Berlin Berlin Germany; ^2^ Department of Cardiology and Pneumology University Medicine Göttingen (UMG) Göttingen Germany; ^3^ Department of Medicine University of Mississippi Jackson MS USA

A significant breakthrough in contemporary cardiometabolic medicine was the finding that some medications to treat type 2 diabetes mellitus (T2DM) are associated with reduced mortality and a lower risk of heart failure (HF) hospitalization when such patients have already established cardiovascular (CV) disease [EMPA‐REG 1]. In the EMPA‐REG OUTCOME trial, including 7020 patients with T2DM, established CV disease (not including HF as qualifying co‐morbidity), and an estimated glomerular filtration rate > 30 mL/min/1.73 m^2^, there was a pronounced reduction in HF hospitalization (hazard ratio 0.65) and CV death (hazard ratio 0.62) with the sodium‐glucose co‐transporter‐2 (SGLT2) empagliflozin compared with placebo.^1^ These benefits were more related to a reduction in incident HF events rather than to any impact on ischaemic vascular outcomes. Importantly, reductions in the risks of CV death with empagliflozin were consistent across the two doses used and the categories of baseline HbA1c and therefore occurred irrespective of glycaemic control.^2^ The results of the EMPA‐REG OUTCOME trial triggered a lively discussion on mechanisms contributing to the beneficial effects on HF outcomes.^3^, ^4^, ^5^, ^6^, ^7^

So far, the ‘*magic bullet*’ responsible for the favourable HF outcomes of empaglifozin has not yet been identified, and most likely, there is no such single mechanism of action that can explain the benefits in its entirety. Promising mechanisms under discussion refer to an improved oxygen supply to the failing heart via an increase of the haematocrit, a metabolic shift towards the consumption of more ketone bodies when other fuels like glucose fail in HF, an unloading of the kidney with a reduction of glomerular pressure and reduced oxygen consumption in the proximal tubule, as well as natriuresis and volume depletion.^3^, ^7^ All these mechanisms may deal with different co‐morbidity aspects of patients with T2DM that lead to the development or deterioration of HF.^8^, ^9^, ^10^, ^11^

An interesting novel mode of action for empaglifozin has now been suggested by Mustroph *et al*.^12^ In this issue of the *ESC Heart Failure*, they show for the first time that empagliflozin potently reduces Ca^2+^/calmodulin‐dependent kinase (CaMKII) activity in isolated failing and non‐failing murine ventricular myocytes. Importantly, empagliflozin also reduced CaMKII‐dependent phosphorylation of the cardiac ryanodine receptor (RyR2) not only in murine but also in failing human ventricular myocytes. This results in a significantly reduced sarcoplasmic reticulum Ca^2+^ leak and improved contractility as measured by increased Ca^2+^‐transient amplitude in murine and human failing ventricular myocytes. These data demonstrate that empagliflozin may be useful in the treatment of pathologies with increased CaMKII activity, such as HF. Does this mean that the magic bullet has now been identified which is responsible for the favourable HF outcomes with empaglifozin in clinical trials? While we would like to congratulate the authors to this new ‘*piece of the puzzle*’, the answer is most likely not. We rather believe in a multifactorial explanation, and for that, many puzzle pieces make the full picture.

But let us have a closer look into empaglifozin itself and how it may differentiate from others in the class of SGLT2 inhibitors. Interestingly, the authors also report^12^ that in contrast to a robust SGLT2 expression in murine kidney, no SGLT‐2 signal was detected in human or mice myocardium. So does SGLT2 expression in the heart matter? And again, the answer is *most likely not*.

There may also be another explanation for the differing effects and side effects of different SGLT2 inhibitors—namely, the specificity of the glifozins to the SGLT2 receptor, the transporter responsible for the majority of glucose reabsorption by the kidney, over its affinity for SGLT1, the transporter responsible for the majority of glucose absorption by the small intestine. This specificity for SGLT2 can vary greatly and is more than 2500‐fold for empaglifozin, 2235‐fold for ertugliflozin, 1200‐fold for dapagliflozin, 200‐fold for canagliflozin, and 20‐fold for sotagliflozin. With a more than 2500‐fold higher affinity to SGLT2 over SGLT1, empaglifozin has the highest selectivity for SGLT2 within the class, which makes empaglifozin stand out within its class^13^, ^14^, ^15^ (*Figure* *1*).

**Figure 1:**
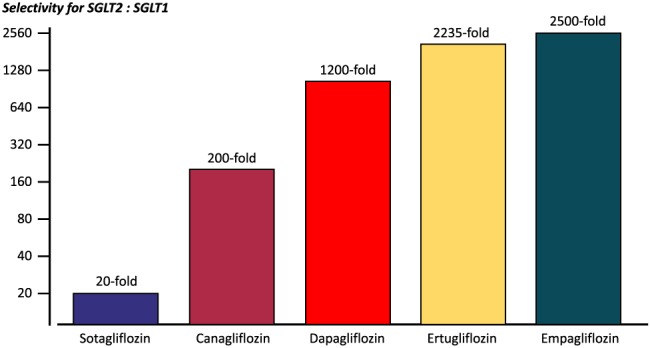
Selectivity of different compounds of the class for the sodium‐glucose co‐transporter‐2 (SGLT2) vs. SGLT1.

So does transporter selectivity matter? Maybe yes but we do not yet know the answer. We only know some pieces of the puzzle but have not yet seen the full picture. Most of the pharmacological effects of SGLT2 inhibitors have the potential to reduce the development and progression of HF. Thus, the potential for benefit with these agents should be properly tested across the spectrum of HF, i.e. in patients with reduced, mid‐range, and preserved left ventricular ejection fraction (LVEF), in randomized controlled trials. Empagliflozin is currently being studied in patients with HF and preserved LVEF (EMPEROR‐Preserved, NCT03057951) and with reduced LVEF (EMPEROR‐Reduced, NCT03057977), including HF patients with and without T2DM. Dapagliflozin is also being studied but only in patients with reduced ejection fraction (Dapa‐HF, NCT03036124). Sotaglifozin is now studied in HF patients after acute worsening of HF, and these patients mostly have reduced LVEF with some added with preserved LVEF (SOLOIST‐WHF; NCT03521934). We are not aware of a study in HF using canagliflozin.

While it is intriguing to speculate about mechanisms of action, the clinical benefits shown in trials are what finally will matter most clinically. By that time when the clinical HF trials will report their results, we can only hope that more pieces of the puzzle will be identified that will explain the ‘why’ and ‘how’ behind the clinical effects seen in T2DM patients where SGLT2 inhibitors are now the mainstay of therapy.

In summary, of the clinically used or currently tested SGLT2 inhibitors, empagliflozin has the highest SGLT2 specificity. Whether this receptor specificity or the previously mentioned CaMKII activity is truly relevant and explains the benefits seen in T2DM, future studies will tell.

## Conflict of interest

S.D.A. reports research support from DZHK Germany, European Union, Vifor International, and Abbott Vascular and fees for trial/registry‐related consultations from Bayer, Boehringer Ingelheim, CVRx, Janssen, Novartis, Servier, and Vifor International. J.B. has received research support from the National Institutes of Health, PCORI, and the European Union and serves as a consultant for Adrenomed, Amgen, Array, AstraZeneca, Bayer, Berlin Cures, Boehringer Ingelheim, Bristol‐Myers Squibb, CVRx, G3 Pharmaceuticals, Innolife, Janssen, Lantheus, LivaNova, Luitpold, Medtronic, Merck, Novartis, Relypsa, Roche, Sanofi, SC Pharma, Vifor, and ZS Pharma.

Both S.D.A. and J.B. serve on the Executive Steering Committee of the EMPEROR trials programme, which is assessing the use of empagliflozin to improve outcomes in patients with heart failure. S.D.A. serves on the Executive Steering Committee of the EMPERIAL trials programme, which is assessing the use of empagliflozin to improve exercise capacity in patients with heart failure.
